# 2-amino-1-methyl-6-phenylimidazo(4,5-b) pyridine (PhIP) induces gene expression changes in JAK/STAT and MAPK pathways related to inflammation, diabetes and cancer

**DOI:** 10.1186/s12986-016-0111-0

**Published:** 2016-08-20

**Authors:** Lora J. Rogers, Alexei G. Basnakian, Mohammed S. Orloff, Baitang Ning, Aiwei Yao-Borengasser, Vinay Raj, Susan Kadlubar

**Affiliations:** 1 University of Arkansas for Medical Sciences, 4301 W Markham St, #580, Little Rock, AR 72205 USA; 2 National Center for Toxicological Research, NCTR Rd, Redfield, AR 72132 USA; 3 Pulaski Technical College, 3000 W. Scenic Dr, North Little Rock, AR 72118 USA

**Keywords:** PhIP, Adipocyte, Gene expression, Inflammation, Diabetes, Cancer risk

## Abstract

**Background:**

2-amino-1-methyl-6-phenylimidazo(4,5-b)pyridine (PhIP), a heterocyclic aromatic amine (HCA) formed in meat that is cooked at high temperatures and then ingested, can potentially be retained in human adipose tissues.

**Methods:**

To determine if PhIP is bioactive in the adipocyte, we exposed a human adipocyte cell line,HepG2 and Caco-2 cells to low dose PhIP. Uptake and retention of PhIP was determined and cytotoxicity was assessed by the TUNEL assay. Relative expression of PhIP-activating genes (CYP1A1, CYP1A2, SULT1A1 and UGT1A1) was determined by RT-PCR and global expression changes were also examined.

**Results:**

The percent retention of 0.1 μCi [^14^C]-PhIP over a 24 h period was significantly higher in the adipocyte than the HepG2 (*p* = 0.0001) and Caco-2 (*p* = 0.0007) cell lines. Cytotoxicity rates were 14.4 and 2.6 % higher compared to controls in Caco-2 and HepG2 cells (*p* < 0.001 and 0.054, respectively); no significant differences were detected in adipocyte cells (*p* = 0.18). Caco-2 and HepG2 cells, respectively, had significantly higher basal expression of CYP1A1 (*p* = 0.001, *p* = 0.003), SULT1A1 (*p* = 0.04, *p* < 0.001) and UGT1A1 (*p* < 0.001, *p* = 0.01) compared to the adipocyte. Exposure to 5nM PhIP did not significantly induce expression of these genes in any of the cell lines. Global gene expression analysis of mature adipocytes exposed to 5nM PhIP for 72 h resulted in statistically significant changes in 8 genes (*ANGPTL2*, *CD14*, *CIDEA*, *EGR1*, *FOS*, *IGFBP5*, *PALM* and *PSAT1*). Gene-gene interaction and pathway analysis indicates that PhIP modulates genes controlled by the STAT3 transcriptional factor and initiates leptin signaling *via* the JAK/STAT and MAPK pathway cascades. Early growth response 1 (EGR1) and prostaglandin synthase 2 (COX-2) were down-regulated *via* c-Fos, while insulin binding protein 5 (IBP5) was up regulated. Expression of transcription factors (ANGPTL2, HP, LEP, SAA1, SAA2), genes related to inflammation (SAA1, LEP), diabetes (IGFBP5) and cancer risk (SAA2) were also elevated upon exposure to 5 nM PhIP..

**Conclusions:**

PhIP mediates gene expression changes within the adipocyte, and the pathways most affected are related to cancer and other chronic diseases. Further studies are needed on the relationship between dietary carcinogens such as PhIP with cancer, obesity and diabetes.

**Electronic supplementary material:**

The online version of this article (doi:10.1186/s12986-016-0111-0) contains supplementary material, which is available to authorized users.

## Background

Heterocyclic aromatic amines (HCAs) are dietary chemicals formed when muscle meat is cooked at high temperatures, such as broiling, frying or grilling. Lipids make up 70 % of white adipose tissue in humans and this endocrine organ represents a reservoir for many lipophilic contaminants [1, 2]. The most abundant lipophilic HCA in cooked meat is 2-amino-1-methyl-6-phenylimidazo[4,5-b] pyridine (PhIP) [3]. PhIP is a procarcinogen that is metabolically activated by cytochromes P450 (CYP) 1A1 and 1A2 to form the metabolite, *N*-hydroxy-PhIP (*N*^2^-OH-PhIP) [4–6]. *N*^2^-OH-PhIP can then undergo detoxification reactions (i.e. glucuronidation) or reactions that generate reactive metabolites (i.e. sulfation) [7–9]. *N*^2^–OH-PhIP metabolites induce chromosomal aberrations [10], form PhIP-DNA adducts in colon mucosa [10], and causes colon and mammary carcinomas in animal models [11–16]. The current consensus in the literature is that consumption of well-done meat is associated with both elevated cancer risk and mortality [17]. However, case control studies examining meat consumption, HCA content and cancer risk lack concordance. Conflicting reports in the literature are almost certainly influenced by the difficulty of quantifying exposures, since HCA content can vary 100-fold depending on cooking temperature [18]. Additionally, the interaction between adiposity, HCA exposure and cancer risk is poorly understood.

Obesity is overtaking under-nutrition and infectious diseases as the most significant causes of ill-health [19]. Obesity is also predicted to have the major influence on cancer incidence, since overweight and obesity are recognized risk factors for various cancers [20]. In addition to its role as an endocrine organ, adipose tissue acts as a reservoir for a large fraction of lipophilic contaminants, including persistent organic pollutants, or POP [21]. A study of the distribution of radiolabeled PhIP in mice demonstrated that after administration of a single dietary equivalent dose of PhIP, the highest concentrations were detected in the stomach, intestine and adipose tissue [22]. Others have reported that human mammary lipid can be mutagenic, and although the identity of the mutagens was not confirmed, the lipid was isolated using a solid-phase tandem extraction procedure originally developed for extraction of heterocyclic amines. The presence of substances in extracts of essentially normal human mammary lipid induced point mutation in bacteria in the presence of metabolizing enzymes and chromosomal damage (signaled by micronucleus formation) in metabolically competent human cells [23].

In the current study, we compared the uptake and retention of PhIP into a human adipocyte cell line (differentiated from human mesenchymal stem cells, (hMSC)), in comparison with a hepatocellular cell line (HepG2) and a colorectal cell line (Caco-2) in order to determine if there were differences. We also compared the ability of PhIP to induce DNA damage and subsequent cell death in these three cell lines. We examined the ability of PhIP to induce gene expression changes in PhIP metabolizing enzymes in each of the cell lines. Finally, global changes in gene expression in the PhIP-exposed adipocyte cell line were also explored.

## Methods

### Cell culture and differentiation

The identity of all cell lines was confirmed by submission to American Type Culture Collection (ATCC) (Manassas, VA) for analysis by short tandem repeat methodology on December 9, 2015. Human Mesenchymal Stem Cells (hMSC) were obtained from AllCells (Emeryville, CA). The hMSCs were expanded in growth medium (GM) consisting of alpha Minimum Essential Medium (αMEM), supplemented with 16.5 % fetal bovine serum (FBS) (Tissue Cultures Biologicals, Long Beach, CA), 2 mM L-glutamine (Sigma-Aldrich, St. Louis, MO), and penicillin/streptomycin at a concentration of 100 μg/ml each (Thermo Fisher Scientific, Watham, MA), in a humidified incubator at 37 °C, with 5 % CO_2_. Cells were seeded 2.0 × 10^5^ cells/well in 6 well plates (for RNA isolation) or 2.0 × 10^4^ /well in 24 well plates (for uptake/retention experiments). Fat differentiation medium (FDM), composed of GM supplemented with 0.5 μM dexamethasone, 0.5 μM isobutylmethylxanthine and 50 μM indomethacin (Sigma-Aldrich, St. Louis, MO), was used to initiate adipocyte differentiation. Fresh FDM was applied every 3-4 days. Differentiation reached a lipid maximum around day 21 at which time PhIP treatments began. Correlation of differentiated vs undifferentiated hMSC was determined by AdipoRed™ Assay Reagent (Lonza Walkersville, Inc. Walkersville, MD) and Trypan Blue (Sigma-Aldrich, St. Louis, MO) readings using a SpectraMax M5 Molecular Devices (VWR, Atlanta, GA) (*R*^*2*^ = 0.9826).

HepG2 and Caco-2 cell lines were purchased from ATCC and maintained following the protocol described by ATCC at 37 °C in an incubator containing 5 % CO2. HepG2 and Caco-2 cells were cultured in EMEM + L-glutamine (ATCC) supplemented with 10 % FBS and EMEM (ATCC) 20 % FBS, respectively. Penicillin/streptomycin at a concentration of 100 μg/ml (Thermo Fisher, Watham, MA) was added to both media.

### Uptake of [^14^C]-PhIP into human cell lines

All cell lines were treated with 5 μM unlabeled PhIP plus 0.1 μCi [^14^C]-PhIP (Toronto Research Chemicals, Toronto, Ontario, Canada) in 0.5 ml FDM using dimethyl sulfoxide (DMSO) (Sigma-Aldrich, St. Louis, MO) as vehicle for all PhIP exposures. The PhIP solution was diluted in culture medium and cells were exposed for 0, 5, 7, 15, 60 and 300 s (s). The cells were then lysed with 100 μl RNAqueous lysis buffer solution (Ambion by Life Technologies Corp., Austin, TX) and the lysate was transferred to a Polyethylene Terephthalate (PET) 24 well sample counting plate containing 300 μl OptiPhase SuperMix scintillation cocktail (Perkins Elmer, Waltham, MA). Sample scintillation counting in corrected cycles per minute (CCPM) was read using the MicroBeta Trilux 1450 LSC and Luminescence Counter (Perkin Elmer, Waltham, MA). Samples were run in triplicate and comparisons between cell types were assessed by t-test.

### Release of [^14^C]-PhIP from human cell lines

Cell lines were plated as described for the uptake studies. Each well was treated with 5 μM unlabeled PhIP plus 0.1 μCi ^14^C-PhIP in FDM using DMSO as vehicle and allowed to reach equilibrium for 30 min. The medium (100 μl) was then transferred to PET 24 well sample counting plates containing 300 μl OptiPhase SuperMix scintillation cocktail for counting. Fresh medium (0.5 ml) was applied to the cells, and then removed after 0**,** 0.5, 1, 2, 5, 15, 60 and 1440 min of exposure. 100 μl aliquots of the fresh medium were transferred to scintillation plates. Cells were then lysed with 100 μl lysis buffer and the lysate was transferred to scintillation plates and CCPM were recorded.

### TUNEL assay

Cells were plated at 1.0 × 10^4^ per well onto Lab-Tek 8 well Chamber Slides (Nalge Nunc International, Rochester, NY) and allowed to grow overnight in a humidified incubator at 37 °C, with 5 % CO_2_. Adipocytes were allowed to differentiate using medium described above for 21 days. Quadruplicate cell samples were exposed to vehicle or PhIP (5 μM) up to 72 h. TUNEL assays were performed using the In Situ Cell Death Detection Kit (Roche Diagnostics, Indianapolis, IN) following the manufacturer’s protocol. A set of short band filters with respective emission and excitation wavelengths were used for the green spectrum (FITC) and blue spectrum (DAPI) for the TUNEL-positive objects and nuclei, respectively.

### Effect of PhIP exposure on expression of PhIP-metabolizing enzymes in human cell lines

Changes in the expression of PhIP- metabolizing enzymes were assessed in HepG2, Caco-2 and hMSC-derived adipocytes seeded at 2 × 10^5^ cells/well in 6- well plates. Medium containing vehicle (DMSO) or PhIP at a final concentration of 5 nM was applied, and the cells were returned to the incubator. All assays were performed in triplicate. Medium with vehicle or PhIP was replaced daily. After 72 h, the cells were washed in ice-cold Dulbecco’s phosphate-buffered saline (DPBS) (Cellgro, Thermo Fisher Scientific, Watham, MA), lysed and total RNA was isolated using the RNAqueous Kit (Ambion). RNA quality and quantity was measured using a NanoDrop^TM^ 8000 Spectrophotometer (Thermo Fisher Scientific, Watham, MA). The resulting RNA absorption 260/280 nm ratio was 2.0, indicating that the RNA prep was pure. cDNA was generated using 1 μg RNA with High Capacity RNA to cDNA Reverse Transcription Reagent (Life Technologies Corp., Austin, TX) and thermo cycler conditions of 37 °C for 60 min, followed by 95 °C for 5 mins using the GeneAMP 7900 PCR System (Applied Biosystems by Life Technologies Corp., Austin, TX). The resulting cDNA was diluted 1:20 prior to use in real-time RT-PCR.

Real-time PCR of target genes known to metabolize PhIP was performed on the 7900HT cycler (Applied Biosystems, by Life Technologies Corp., Austin, TX) under the following conditions: 95 °C for 10 min, 40 cycles of 95 °C for 1 s, followed by 60 °C for 20 s. A dissociation cycle of 95 °C for 15 s, 60 °C for 15 s and 95 °C for 15 s was performed at the end of each PCR reaction to ensure the amplicon specificity. Fast SYBR Green PCR Master Mix (Applied Biosystems, by Life Technologies Corp., Austin, TX) was used along with 5 μM of primer purchased from Integrated DNA Technologies (Coralville, IA). Primer sequences are available in Table 1. Expression was normalized to the endogenous control reference gene *HRPT1. HRPT1* was identified as “Best Gene” by Normfinder software [24] using gene stability values compared with β-Actin and GAPDH in all three cell types over treatments. Expression was quantified using the relative gene expression 2 ^-∆∆CT^ method [25].

**Table 1:** Primer sequences

Symbol	Forward Primer	Reverse Primer	Accession number
CYP1A1	GATCCCAGGCTCCAAGAGTC	ATCTTGGAGGTGGCTGAGGTA	NM_000499
CYP1A2	CACCTGCCTCTACAGTTGGTA	GAAGCTCTGTGGCCGAGAAG	NM_000761
SULT1A1	AGGAGTTCATGGACCACAGC	TGAAGGTGGTCTTCCAGTCC	NM_177536.2
UGT1A1	AATAAAAAAGGCTCTGCT	ACATCAAACTGCTTTCTGC	NM_000463
HPRT1	CTCCTGAGCAGTCAGCCCGC	CACTAATCACGACGCCAGGGCT	NM_000194

Primer Sequences of PhIP metabolizing enzymes

### Global gene expression analysis

hMSC cells were differentiated and exposed to varying concentrations of PhIP for 72 h. RNA was isolated by the method described above. RNA integrity was analyzed on an Agilent 2100 Bioanalyzer (Agilent, Santa Clara, CA, USA). Total RNA (500 ng) was used to generate cRNA for hybridization with the Illumina TotalPrep™ RNA Amplification Kit (Life Technologies) according to the manufacturer’s instructions. Microarray analysis was performed at the University of Arkansas for Medical Sciences Genomics Core. 750 ng cRNA was used for hybridization to Illumina HumanHT-12_v4_BeadChip for Gene Expression according to the manufacturer’s protocol. BeadChips were scanned with the Illumina iScan system, and data collected using GenomeStudio software v2011.1. Data was generated using the gene expression module of Illumina Genome Studio software after quality control analysis. The expression intensities were median-normalized after log transformation. Differentially expressed genes were determined using an empirical Bayes test implemented in Linear Models for Microarray Data (Limma) package. A fold-change > 1.5 and p-value < 0.05 were chosen as the cutoff criteria for screening differentially expressed genes. The p-values were adjusted for multiple testing by controlling the false discovery rate according to the Benjamini Hochberg method. All analyses were conducted using the R statistical environment (R Foundation for Statistical Computing, Vienna, Austria).

### Pathway analysis

Functional profiling of differentially affected biological processes, functions, pathways, and networks for the differentially expressed genes (DEGs) were evaluated using open source tools and the commercial pathway packages Metacore (MetacoreTM, www.genego.com), DAVID and STRING databases. Differentially expressed transcripts were mapped to the top most differentially expressed pathways and process networks. For network analysis of differentially expressed genes, Metacore was employed to identify any direct interactions amongst the DEGs. STRING database was also used to explore the gene-gene interactions based on experimental evidence and predictions based on homology, text mining, and co-occurrence.

### Statistical analysis

Uptake and retention data was reported as the percent of total [^14^C]-PhIP that remained in cell lysate at various time points. Percent means ± SD of triplicate measurements and unpaired Student t-tests were employed to identify significant differences between the cell lines. Differential gene expression was determined by the 2^-∆∆CT^ method [24] and reported as mean ± SD of triplicate measurements. The comparisons of significance were carried out by unpaired Student’s *t*-test. Cytotoxicity was assessed by unpaired t-tests

## Results

### Uptake and retention of [^14^C] Up-PhIP in cell lines

**Fig. 1:**
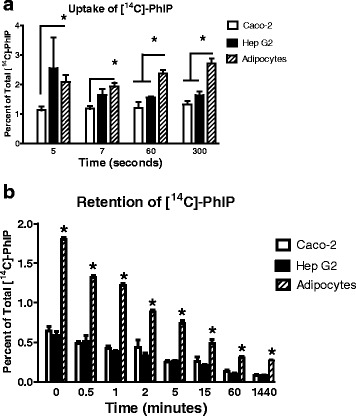
**a** Uptake of 5 mM PhIP + 0.1 mM [14C]-PhIP in Caco-2, HepG2 and adipocytes. (*p < 0.05) (**b**) Retention of 5 mM PhIP + 0.1 mM [14C]-PhIP in Caco-2, HepG2 and adipocytes. Data presented as Mean ± SD. (*p < 0.05 across time course)

Uptake of [^14^C]-PhIP into Caco-2, HepG2 and hMSC derived adipocyte (at 70 % differentiation) cells was rapid in all cell lines. Maximal uptake of [^14^C]-PhIP was apparent after 5 s of exposure in HepG2 and Caco-2 cell lines and remained unchanged over the time course of the experiment. Overall, Caco-2 and HepG2 cells did not differ significantly. Uptake of [^14^C]-PhIP into adipocytes was significantly higher than into Caco-2 and HepG2 cells at 60 s and 300 s, respectively, and did not appear to plateau throughout the time course of the experiment (Fig. 1a). When levels of [^14^C]-PhIP retained in the cell lines were examined, adipocyte cell [^14^C]-PhIP content was significantly higher than in Caco-2 and HepG2 cells at all of the time points (Fig. 1b).

### Cytotoxicity of PhIP to human cell lines

**Fig. 2:**
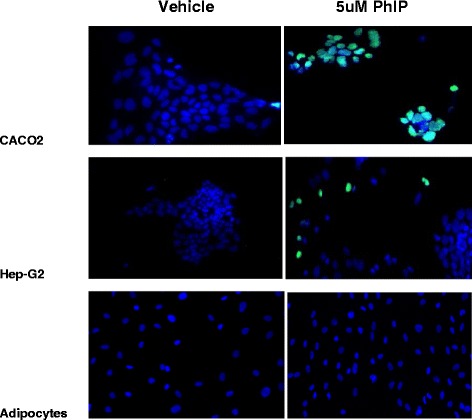
TUNEL assay of Vehicle or 5 μM PhIP exposure for 72 h cytotoxic damage in (**a**) Caco-2 (14.4 % higher than controls, *p* = 0.0019) (**b**) HepG2 (2.6 % higher than controls *p* = 0.0536) and (**c**) adipocyte (no differences from controls, *p* = 0.1790). Green spectrum are TUNEL positive objects, blue spectrum are nuclei

PhIP and other HCAs are genotoxic and cytotoxic at high doses and induction of cell death (apoptosis) is cell-type specific [26–28]. In order to determine if PhIP had adverse effects at low concentrations and in adipocytes, we exposed Caco-2, HepG2 and adipocyte cells to 5 μM PhIP for 72 h. Irreversible cell death associated with genotoxic DNA fragmentation was assessed by the TUNEL assay. Figure 2 is a representative area of the slides used by TUNEL assay for analysis (Fig. 2a = Caco-2 cells, Fig. 2b = HepG2 cells and Fig. 2c = Adipocyte cells). Unpaired t-test revealed statistical differences from controls (*p* < 0.05) in the Caco-2 cells at 14.4 % (*p* = 0.002) and borderline significance in the HepG2 cells at 2.6 % (*p* = 0.054). There were no significant differences between control and PhIP-treated adipocyte cells (*p* = 0.18).

### Relative expression of PhIP-metabolizing enzymes in cell lines

**Fig. 3:**
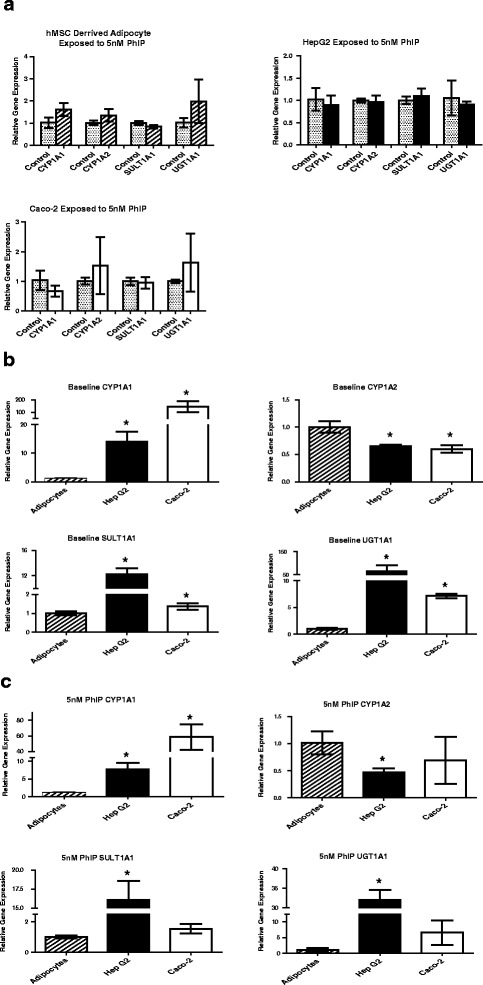
**a** Relative gene expression of PhIP metabolizing enzymes compared to controls after exposure to 5 nM PhIP for 72 h. Data is presented as Mean ± SD *p < 0.05. Expression of CYP1A1 (*p* = 0.0511, *p* = 0.5459, *p* = 0.1615), CYP1A2 (*p* = 0.1194, *p* = 0.6966, *p* = 0.4045), SULT1A1 (*p* = 0.0866, *p* = 0.4488, *p* = 0.6952) and UGT1A1 (*p* = 0.1719, *p* = 0.5599, *p* = 0.3261) in Adipocyte, HepG2 and Caco-2 cells respectively. There were no significant differences from controls. **b** Relative gene expression of baseline PhIP metabolizing enzymes normalized to adipocyte controls. Data is presented as Mean ± SD *p < 0.05. Expression of CYP1A1 (*p* = 0.0030, *p* = 0.0055), CYP1A2 (*p* = 0.0044, *p* = 0.0047), SULT1A1 (*p* = 0.0001, *p* = 0.0358) and UGT1A1 (*p* = 0.0104, *p* = 0.0001) in HepG2 and Caco-2 cells respectively. HepG2 and Caco-2 cells had significantly higher baseline PhIP metabolizing enzymes than adipocytes with the exception of CYP1A2. **c** Relative gene expression of PhIP metabolizing enzymes after 5nM PhIP exposure, normalized to adipocyte treatment. Data is presented as Mean ± SD *p < 0.05. Expression of CYP1A1 (*p* = 0.0030, *p* = 0.0037), CYP1A2 (*p* = 0.0140, *p* = 0.3050), SULT1A1 (*p* = 0.0005, *p* = 0.0516) and UGT1A1 (*p* = 0.0001, *p* = 0.0754) in HepG2 and Caco-2 cells respectively. HepG2 cell expression was significantly higher than the adipocyte in all four PhIP metabolizing enzymes while Caco-2 was significantly higher in the CYP1A1 enzyme only

Since low level PhIP exposure did not induce cell death in the adipocyte cell line, but did in Caco-2 and HepG2 cells, we then examined the expression levels of genes involved in the metabolic activation of PhIP, and determined if exposure of the cell lines to PhIP could induce the expression of these genes. Exposure of each cell line to 5 nM PhIP did not significantly increase the expression of PhIP-metabolizing genes in any cell line tested (Fig. 3a). We then compared baseline levels of CYP1A1, CYP1A2, SULT1A1 and UGT1A1 in adipocytes, HepG2 and Caco-2 cell lines. Expression of CYP1A1 was 10- and 100-fold higher in HepG2 and Caco-2 cell lines compared to adipocytes, where expression of CYP1A1 was negligible. CYP1A2 expression was negligible in all cell lines, while SULT1A1 and UGT1A1 were most highly expressed in HepG2 cells (Fig. 3b). Exposure of the cell lines to 5 nM PhIP did not result in any changes in this observed pattern (Fig. 3c).

### Global gene expression changes in h-MSC-derived adipocytes exposed to PhIP

PhIP was taken up and retained to a greater degree by adipocytes compared to HepG2 and Caco2; thus, we sought to determine whether PhIP within the adipocyte cell line remained inert, or exhibited biological effects. Others have reported interactions of PhIP with both the androgen and estrogen receptor at physiologically achievable concentrations to induce the expression of steroid-responsive genes [29–31]. Therefore, we then examined global gene expression in mature adipocytes. We exposed mature adipocytes to varying concentrations of PhIP (5 nM – 2.5 μM) for 72 h. Exposure to 5nM PhIP resulted in significant differential expression of 64 genes in an unadjusted model (Additional file 1: Table S1, see end of document). After adjustment for multiple comparisons, 8 genes (*ANGPTL2*, *CD14*, *CIDEA*, *EGR1*, *FOS*, *IGFBP5*, *PALM* and *PSAT1*) remained significant at the *p* = 0.05 level (Table 2). Of the eight genes, four of them (*ANGPTL2*, *EGR1*, *FOS* and *IBP5*) persisted to be important in the gene-gene interaction analysis.

**Table 2:** Differential expression of genes in h-MSC-derived adipocytes exposed to PhIP

Gene Symbol	Accession	Ave Expression	FC	*P*.Value	adj.*P*.Val
ANGPTL2^a^	NM_012098.2	8.578	1.88	6.19E-07	0.59E-04
EGR1^a^	NM_001964.2	8.750	-2.93	2.65E-10	1.25E-05
FOS^a^	NM_005252.2	7.120	-1.95	2.57E-08	0.61E-05
IBP5^a^	NM_000599.2	8.550	1.91	2.45E-06	0.014
CIDEA	NM_001279.2	6.697	1.49	3.24E-07	0.39E-04
PALM	NM_002579.2	8.575	1.63	4.06E-06	0.018
PSAT1	NM_021154.3	8.614	-1.62	4.26E-06	0.018
CD14	NM_001040021.1	7.650	1.58	1.93E-05	0.05

Differentially expressed genes in hMSC derived adipocyte exposed to 5 nM PhiP for 72 h, adjusted for multiple comparisons, significant at the *p* = 0.05 level

**Fig. 4:**
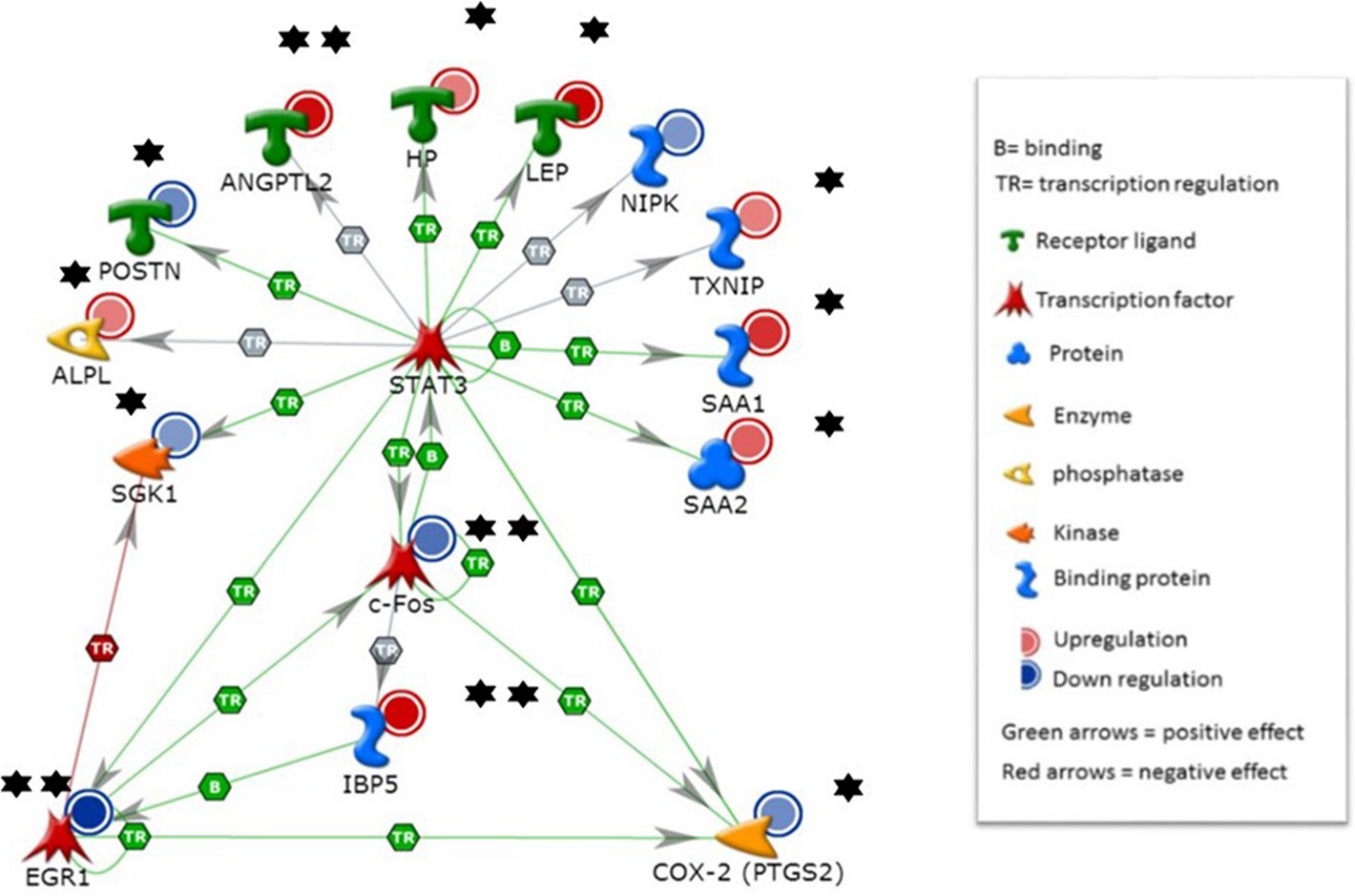
5 nM PhIP activates STAT3-mediated transcriptional regulation. Genes within the STAT3 pathway were modulated by exposure to 5 nM PhIP for 72 h. One star denotes significance at <0.05 in the unadjusted model and two stars denote significance at <0.05 after adjustment for multiple comparisons

**Fig. 5:**
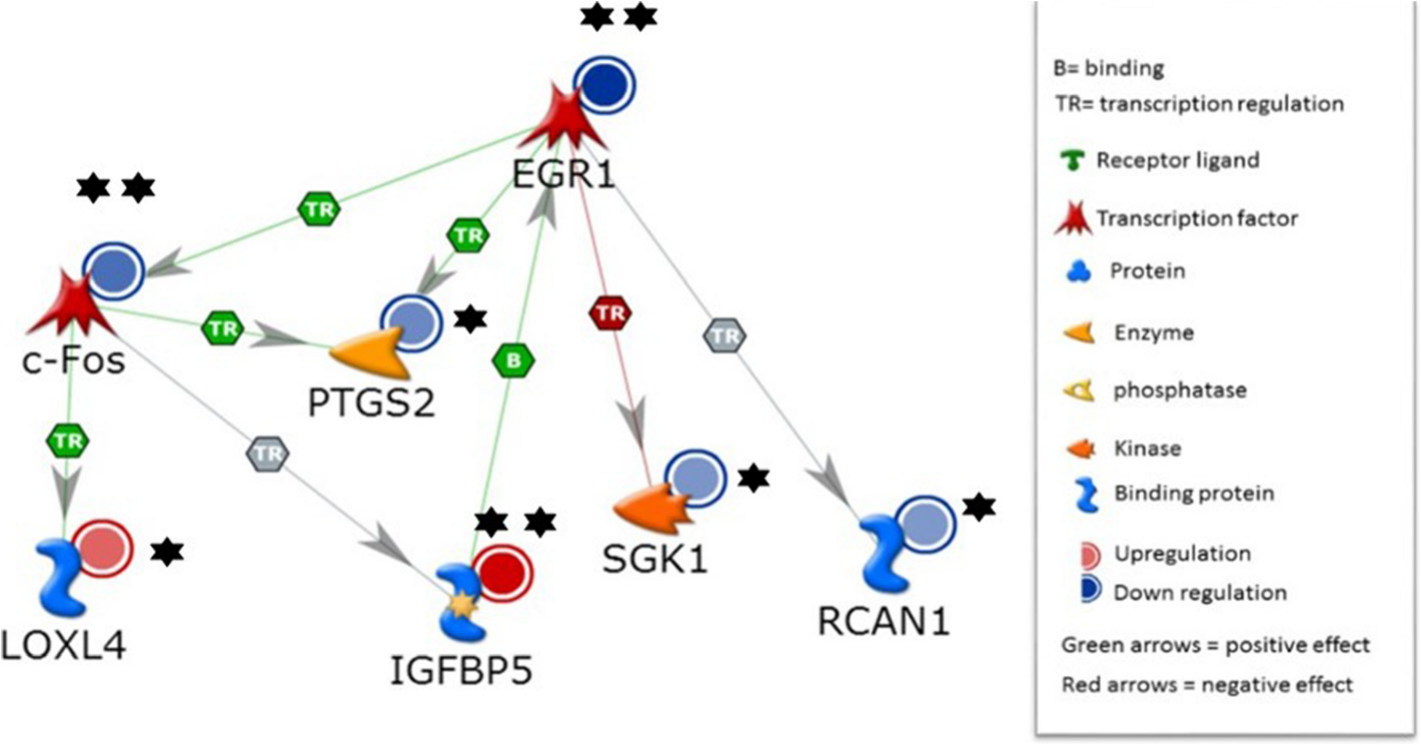
Direct interactions between genes induced or suppressed by 5nM PhIP exposure. One star denotes significance at <0.05 in the unadjusted model and two stars denote significance at <0.05 after adjustment for multiple comparisons

^a^Receptor ligands, transcriptional factors and/or binding proteins that are significant and are controlled by the STAT3 transcriptional factor in the gene-gene interaction and pathway analyses (see Figs. 4 and 5)

### Gene-gene interaction and pathway analysis

To functionally validate the roles of these genes in biological pathways, the top significantly induced genes compared to vehicle-treated controls (at *p* = 0.01) in the unadjusted model were then subjected to gene-gene interaction and pathway analysis. From this analysis, we found that PhIP modulated genes controlled by the STAT3 transcriptional factor (Fig. 4). Among genes controlled by STAT3, exposure to PhIP resulted in the down-regulation of early growth response 1 (EGR1) and prostaglandin-endoperoxide synthase 2 (PTGS2) *via* c-Fos, while insulin binding protein 5 (IBP5) was up regulated. The expression of other transcription factors (ANGPTL2, HP, LEP, SAA1, and SAA2) were up-regulated by PhIP exposure. When direct interactions were assessed, two genes were found to be down-regulated (i.e. cFos, EGR1; *p* = 0.61E-05 and 1.25 E-05, respectively), and IBP5 was upregulated (*p* = 0.014). Analysis also revealed two genes that are part of the gene-gene interaction network, which are predicted to be down regulated (i.e. PTGS2 and SGK1). Through gene-gene interaction analysis we were able to reveal that the down-regulated transcriptional factors cFOS and EGR1 that are downstream of MAPK signaling display direct physical DNA-DNA interaction as predicted by Metacore software (Fig. 5) and elsewhere.

## Discussion

This is the first report of PhIP mediating changes in gene expression within the adipocyte. Along with other heterocyclic amines, PhIP is lipophilic [22]. We hypothesized that PhIP would be preferentially taken up by the adipocyte and associate with the lipid droplet and not undergo metabolic activation that leads to DNA damage. In agreement with this hypothesis, PhIP in fact is not activated in the adipocyte and does not cause cell death measurable by TUNEL. Additionally, the adipocyte cell line employed in these studies had negligible expression of the enzymes necessary for metabolic activation, and exposure to PhIP did not induce their expression. However, we did find that PhIP induces changes in gene expression in various genes that are related to inflammation (SAA1, LEP), diabetes (IBP5) and cancer risk (SAA2).

Reports in the literature support the role of adipose tissue as sites of interaction with environmental chemicals. It has long been recognized that persistent organic pollutants (POPs) are sequestered in adipose tissue, and this sequestration offers initial protection of other organs and tissues from the harmful effects of these chemicals. Over time, however, adiposity can increase the body burden of POPs; weight loss and normal lipolysis can then release these chemicals, resulting in tissue and organ damage. Adipose is also a target of POPs and is thought to participate in the mediation of the metabolic effects of POPs (reviewed in [32]). In addition to POPs, there are recent reports that Bisphenol-A (BPA), a known endocrine disrupting chemical, can regulate the expression of genes within adipocytes that participate in metabolic homeostasis and obesity [33]. Efforts to limit exposures to these chemicals have received much attention. There have also been efforts to reduce the consumption of red and processed meats based on their association with cancer, cardiovascular disease and diabetes [34]. And while obesity is a risk factor for these diseases as well, the potential effect of food-borne HCAs such as PhIP on adipocyte function has been unrecognized. The observation that PhIP induces gene expression changes in important pathways could have profound implications in human health, particularly in Western countries where the consumption of well-done cooked meat is common. And analysis of world meat consumption patterns documents a surge in meat consumption by emerging countries such as China and Brazil [35].

Analysis of global gene expression revealed that low levels of PhIP perturbed pathways related to obesity, inflammation, diabetes and risk of certain cancers. For example, cFOS positively binds and activates STAT3, which positively activates EGR1, cFOS, and PTGS2 (COX-2). PhIP exposure suppressed this pathway, while it induced the expression of IBP5, a biomarker of obesity [36, 37], suggesting that the effects of PhIP exposure are complex. However, STAT3 can physically bind to and positively activate serum amyloid A1 binding proteins A1 and A2 (SAA1, SAA2) and these proteins were up-regulated by PhIP exposure. SAA1 encodes a member of the serum amyloid A family of apolipoproteins, which is a major acute phase protein that is highly expressed in response to inflammation and tissue injury [38, 39]. SAA1 also plays an important role in high density lipoprotein (HDL) metabolism and cholesterol homeostasis [40]. High levels of this protein are associated with chronic inflammatory diseases [38]. SAA2 may also be a potential biomarker for certain tumors [41, 42]. In the present study, these genes were significantly modulated in the unadjusted models, and some retained significance when adjusted for multiple comparisons. Future studies in our laboratory will explore the impact of PhIP exposure under varied experimental conditions to determine the exact contributions of pathway genes to the effects of PhIP on adipocytes.

The magnitude of changes in gene expression was modest in this report, but a strength of the current study is that the PhIP concentrations examined approach dietary relevance in humans. PhIP daily intake estimates range from 0 to 630 ng/day according to meat-specific food frequency questionnaires (FFQs) and diet diaries coupled with the NCI Computerized Heterocyclic Amines Resource for Research in Epidemiology of Disease (CHARRED), [43, 44]. By examining low doses of PhIP, we more closely approach levels that could be expected in human populations. While we only reported on the effects of 5 nM PhIP, concentrations ranging up to 100 μM were also examined. Interestingly, as PhIP concentrations increased, the numbers of genes and pathways that were significantly changed decreased. This suggests that in vitro studies employing high, non-physiological concentrations may not identify important interactions. We also measured changes that occurred after relatively short time intervals; effects of long-term, low-dose exposures are unclear and are the subject of further investigation in our laboratory.

Nonetheless, the observation that PhIP is mediating gene expression changes within the adipocyte, and that the pathways most affected are related to cancer and other chronic disease is intriguing. The established paradigm of PhIP-induced carcinogenesis involves metabolic activation [45], DNA adduct formation resulting in deleterious mutations that initiate carcinogenesis [28, 46–48]. However, there are reports in the literature of PhIP interacting with both the estrogen and androgen receptor at physiologically achievable concentrations [29–31], indicating that DNA adduct formation is only one pathway of PhIP effects, and perturbation of the microenvironment produced by adiposity could generate changes conducive to cancer and other chronic diseases.

## Conclusions

The observation that PhIP is mediating gene expression changes within the adipocyte, and that the pathways most affected are related to cancer and other chronic disease suggests studies that address mechanisms other than DNA adduct formation are needed to clarify the relationship between dietary carcinogens such as PhIP with cancer, obesity and diabetes.

## Abbreviations

ATCC, American Type Culture Collection; Caco-2, colorectal cell line; CCPM, corrected cycles per minute; CHARRED, computerized heterocyclic amines resource for research in epidemiology of disease; CYPs, cytochrome P450s; DEGs, differentially expressed genes; DMSO, dimethyl sulfoxide; EGR1, early growth response 1; FBS, fetal bovine serum; FDM, fat differentiation medium; FFQs, food frequency questionnaires; GM, growth medium; HCAs, heterocyclic aromatic amines; HDL, high density lipoprotein; HepG2, hepatocellular cell line; hMSC, human mesenchymal stem cells; HP, haptoglobin; IBP5, insulin binding protein 5; IGFBP5, insulin-like growth factor binding protein 5; LEP, leptin; Limma, linear models for microarray data; N^2^-OH-PhIP, N-hydroxy-PhIP; NAT, N-acetyltransferase; NMT, N-methyltransferase; PET, polyethylene terephthalate; PhIP, 2-amino-1-methyl-6-phenylimidazol[4,5-b]pyridine; POP, persistent organic pollutants; PTGS2, prostaglandin-endoperoxide synthase 2; SAA1, SAA2, serum amyloid A1 binding proteins A1 and A2; SULTs, sulfotransferase; UGTs, glucuronidation; αMEM, alpha minimum essential medium

## Additional file

Additional file 1: Table S1. Global gene expression changes in h-MSC-derived adipocytes exposed to PhIP. (DOCX 24 kb)
