# Supplementary material for: 2-amino-1-methyl-6-phenylimidazo(4,5-b) pyridine (PhIP) induces gene expression changes in JAK/STAT and MAPK pathways related to inflammation, diabetes and cancer
Source: Nutr Metab (Lond). 2016 Aug 20;13:54. doi: 10.1186/s12986-016-0111-0 (PMC4992261; doi:10.1186/s12986-016-0111-0)
Supplement: Additional file 1: Table S1. — Global gene expression changes in h-MSC-derived adipocytes exposed to PhIP. (DOCX 24 kb) [file 12986_2016_111_MOESM1_ESM.docx]

**Additional file 1: Table S1.**

**Global gene expression changes in h-MSC-derived adipocytes exposed to PhIP**

| **Symbol** | **Accession** | **AveExpr** | **Fold Change** | **P.Value** | **Adj.P.Val** |
| --- | --- | --- | --- | --- | --- |
| ACTC1 | NM_005159.4 | 8.370 | 1.5 | 0.009741 | 0.583524 |
| ALPL | NM_000478.3 | 9.923 | 1.5 | 0.000191 | 0.123839 |
| **ANGPTL2** | **NM_012098.2** | **8.578** | 1.9 | **6.19E-07** | **0.005858** |
| C10orf116 | NM_006829.2 | 8.828 | 1.7 | 7.25E-05 | 0.100325 |
| C1QTNF1 | NM_198594.1 | 9.510 | -1.6 | 0.000452 | 0.179913 |
| C7orf10 | NM_024728.1 | 8.718 | -1.5 | 0.00288 | 0.407701 |
| **CD14** | **NM_001040021.1** | **7.650** | **1.6** | **1.93E-05** | **0.053812** |
| **CIDEA** | **NM_001279.2** | **6.697** | **1.5** | **3.24E-07** | **0.003858** |
| CLEC3B | NM_003278.1 | 7.755 | 1.6 | 6.18E-05 | 0.096376 |
| CORIN | NM_006587.2 | 9.889 | 1.6 | 0.000109 | 0.10962 |
| CTHRC1 | NM_138455.2 | 9.391 | -1.5 | 0.006469 | 0.517997 |
| **EGR1** | **NM_001964.2** | **8.750** | **-2.9** | **2.65E-10** | **1.25E-05** |
| FAM63A | NM_018379.3 | 7.484 | -1.5 | 0.005249 | 0.489908 |
| FCAR | NM_133279.1 | 7.660 | -1.5 | 0.006722 | 0.522375 |
| **FOS** | **NM_005252.2** | **7.120** | **-2.0** | **2.57E-08** | **0.000608** |
| GRIPAP1 | NM_207672.1 | 9.027 | -1.6 | 0.004701 | 0.479441 |
| HCG2P7 | NR_001318.1 | 10.308 | -1.5 | 0.009844 | 0.585127 |
| HP | NM_005143.2 | 9.735 | 1.5 | 0.005184 | 0.487776 |
| IFITM1 | NM_003641.3 | 7.686 | 1.5 | 0.000151 | 0.117039 |
| **IBP5** | **NM_000599.2** | **8.550** | **1.8** | **2.45E-06** | **0.014484** |
| KIAA1881 | XM_001130790.1 | 9.906 | 1.5 | 0.002197 | 0.37124 |
| *LEP* | *NM_000230.1* | *10.057* | *1.9* | *5.24E-05* | *0.08861* |
| LIMCH1 | NM_014988.1 | 8.792 | 1.6 | 0.002031 | 0.356639 |
| LOC100129362 | XM_001721430.1 | 10.314 | -1.5 | 0.006683 | 0.521814 |
| LOC100132761 | XM_001716956.1 | 8.120 | 2.1 | 0.000189 | 0.123839 |
| *LOC389286* | *NM_001018022.1* | *7.406* | *1.6* | *5.03E-05* | *0.08861* |
| LOC728620 | XR_037241.1 | 10.950 | -1.6 | 0.002525 | 0.39357 |
| LOXL4 | NM_032211.6 | 9.274 | 1.6 | 0.000134 | 0.117039 |
| LRRFIP1 | NM_004735.2 | 8.673 | -1.9 | 0.001052 | 0.269978 |
| MGP | NM_000900.2 | 7.700 | -1.6 | 0.000225 | 0.128608 |
| MIR1974 | NR_031738.1 | 6.948 | 1.7 | 0.000471 | 0.185939 |
| MYL12A | NM_006471.2 | 11.986 | -1.7 | 0.001933 | 0.352611 |
| PABPC1 | NM_002568.3 | 8.429 | -2.1 | 0.000256 | 0.135959 |
| **PALM** | **NM_002579.2** | **8.575** | **1.63** | **4.06E-06** | **0.018343** |
| PCSK5 | NM_006200.2 | 8.403 | 1.5 | 0.000241 | 0.13095 |
| PHGDH | NM_006623.2 | 9.398 | -1.5 | 0.001396 | 0.314551 |
| PHLDA1 | NM_007350.3 | 7.926 | -1.6 | 0.000163 | 0.119865 |
| PLS3 | NM_005032.3 | 9.597 | -1.5 | 0.003067 | 0.409954 |
| POSTN | NM_006475.1 | 8.353 | -1.7 | 0.003498 | 0.427756 |
| PRC1 | NM_199413.1 | 8.372 | -1.7 | 9.80E-05 | 0.105411 |
| **PSAT1** | **NM_021154.3** | **8.614** | **-1.6** | **4.26E-06** | **0.018343** |
| PTGS2 | NM_000963.1 | 9.395 | -1.6 | 0.00015 | 0.117039 |
| QRFPR | NM_198179.2 | 8.969 | -1.5 | 0.006698 | 0.521814 |
| RCAN1 | NM_203417.1 | 9.026 | -1.5 | 0.009527 | 0.58181 |
| RPS4X | NM_001007.3 | 10.592 | 1.5 | 0.002414 | 0.385496 |
| SAA1 | NM_000331.3 | 8.842 | 1.8 | 0.00013 | 0.117039 |
| SAA2 | NM_030754.2 | 7.393 | 1.6 | 9.28E-05 | 0.104511 |
| SEMA3E | NM_012431.1 | 8.575 | -1.8 | 0.001513 | 0.325405 |
| SGK1 | NM_005627.3 | 8.151 | -1.5 | 0.00155 | 0.328969 |
| SHROOM4 | NM_020717.2 | 8.088 | -1.6 | 0.00288 | 0.407701 |
| SLC7A5 | NM_003486.5 | 8.126 | -1.6 | 4.24E-05 | *0.087405* |
| SPARCL1 | NM_004684.3 | 7.609 | -1.6 | 0.000111 | 0.10962 |
| SRPX2 | NM_014467.2 | 8.967 | -1.5 | 0.000266 | 0.13839 |
| STMN2 | NM_007029.2 | 9.712 | 1.5 | 0.005679 | 0.501354 |
| SVEP1 | NM_153366.2 | 9.541 | 1.5 | 0.001485 | 0.323808 |
| THRSP | NM_003251.2 | 7.524 | 1.5 | 0.001291 | 0.299556 |
| TMTC3 | NM_181783.1 | 7.524 | 1.5 | 4.90E-05 | 0.08861 |
| TNS3 | NM_022748.10 | 10.595 | 1.6 | 0.000327 | 0.164949 |
| TOP2A | NM_001067.2 | 8.392 | -1.5 | 0.003062 | 0.409954 |
| TRIB3 | NM_021158.3 | 8.001 | -1.5 | 0.000128 | 0.117039 |
| TXNIP | NM_006472.2 | 12.215 | 1.5 | 9.66E-05 | 0.105411 |
| TYMS | NM_001071.1 | 8.784 | -1.5 | 8.03E-05 | 0.100325 |
| UBE2C | NM_181800.1 | 8.330 | -1.6 | 0.000149 | 0.117039 |
| ZNF394 | NM_032164.2 | 9.369 | -1.6 | 0.009431 | 0.581155 |

Significant differential expression of 64 genes in an unadjusted mode in adipocyte cell line exposed to 5 nM PhiP for 72 hours. Bold text are significantly differentiated after adjustment for multiple comparisons
